# Refracture of a Leg, to Improve Defective Surgery

**Published:** 1851-02

**Authors:** 


*Refracture of a Leg, to Improve Defective Surgery.* By R. D. Mussey, M. D., Prof. of Surgery, Medical College, Ohio.

On the 29th January, 1848, Miss J. E. Kingsley, a school teacher, in Jefferson Co., East Tennessee, in descending a hill, was thrown from a buggy, and had both bones of the left leg broken in two places; one three and a half inches below the knee, the other two and a half inches above the ankle.

It was six weeks before Miss K. began to sit up in bed, and four months before she was able to ride out. She came to Cincinnati in July of the same year. Ever since the injury, the leg had been considerably swollen, and there had not been a day without more or less pain, sometimes severe, extending from the upper fracture to the heel, back of the foot and toes, indicating lesion or compression of the fibular nerves.

Both fractures were firmly consolidated. The lower fracture was well enough, exhibiting no deformity—at the upper one, the leg was sadly bent, exhibiting a prominent external convexity, or angle, so great as to shorten the distance from the knee to the inside of the foot about an inch and a half; the planter surface of the foot looking inward, and its outer edge looking directly downward. Of course the limb was altogether useless in walking; any attempt to apply the foot to the ground aggravating the pain. It was impossible to place the sole of the foot down flat, or bring the heel within an inch of the ground. The limb was therefore left to swing, while Miss K. moved about upon the other leg, and a pair of crutches.

In September, 1848, aided by my son, Dr. Wm. H. Mussey, I operated in the following manner. A firm pad an inch and a half thick was laid upon the inside of the knee, another upon the inside of the ankle, extending five inches up the leg. A splint of hard wood, one inch thick, and three inches wide, was laid, and secured by a bandage upon these pads. A broad padded belt was placed over the angular projection of the fracture, and gradually tightened by a mechanical power, derived from Jarvis' adjuster, till the fracture was crushed, and the leg straightened.

Miss K. having been placed under the influence of chloroform, was wholly unconscious of pain during the operation, and occupied herself all the while, in singing sacred songs, and holding celestial conversation; and while a bandage and splint were being applied to maintain the new position of the limb, finding herself coming to earth again, she entreated most earnestly for more chloroform, to prolong the ecstatic illusion. After the operation, the pain in the leg and foot were diminished, and in two months the fracture was consolidated.

Dec. 12. There is now no pain at the heel, and comparatively little in the leg and foot. The limb has its natural direction, is as long, and apparently as strong as the other. She can now walk with a cane, and limpingly without one.

Feb. 1849. Miss K. now walks very well without crutch or cane, and only now and then feels slight pain in the leg, the nervous injury having been almost repaired. Some months after the above date we saw Miss K. walking well in the street as if nothing had happened.

Cincinnati, Nov., 1850.—*Western Lancet.*

